# Mitochondrial DNA mutation load in a family with the m.8344A>G point mutation and lipomas: a case study

**DOI:** 10.1002/ccr3.1096

**Published:** 2017-11-02

**Authors:** Tina Dysgaard Jeppesen, Noor Al‐Hashimi, Morten Duno, Flemming Wibrand, Grete Andersen, John Vissing

**Affiliations:** ^1^ Copenhagen Neuromuscular Clinic Rigshospitalet University of Copenhagen Copenhagen Denmark; ^2^ Department of Clinical Genetics Rigshospitalet University of Copenhagen Copenhagen Denmark

**Keywords:** Lipomas, mitochondrial myopathy, mtDNA mutation, segregation

## Abstract

Studies have shown that difference in mtDNA mutation load among tissues is a result of postnatal modification. We present five family members with the m.8344A>G with variable phenotypes but uniform intrapersonal distribution of mutation load, indicating that there is no postnatal modification of mtDNA mutation load in this genotype.

## Introduction

Clinical presentation of patients with mitochondrial DNA (mtDNA) mutation differs tremendously. Thus, even patients harboring the same genotype can present with symptoms ranging from monosymptomatic muscle weakness to multiorgan involvement. The clinical variety is, at least in part, a result of differences in mtDNA mutation load among tissues. Findings of high mtDNA mutation load in postmitotic tissues and low levels in highly mitotic tissues 1, 2 has prompted the idea, that there is postnatal modification of mtDNA mutation load depending on the mitotic status of the tissue.

The m.8344A>G point mutation is generally associated with the clinical phenotype myoclonus epilepsy and ragged‐red fibers (MERRF) 3. However, clinical presentation in this patient group varies 4 and symptoms such as hearing impairment, migraine, and psychiatric disorders may be just as common 5, and clinical case reports have even associated the m.8344A>G genotype with a phenotype of multiple lipomas 6, 7, 8, 9, 10, 11, 12, 13, 14, 15. We report the clinical, physiological, morphological, and biochemical findings along with mtDNA mutation load in both mitotic and postmitotic tissues in five family members with the m.8344A>G point mutation lipomas in two. We found a uniform intrapersonal distribution of mutation level and the mtDNA mutation load did not correlate with age. These findings indicate, that there is no postnatal modification of mutation in patients with the m.8344A>G as it is seen in patients with other common point mutation of mtDNA 1, 2.

## Case Presentation

### Subjects

**Figure 1:**
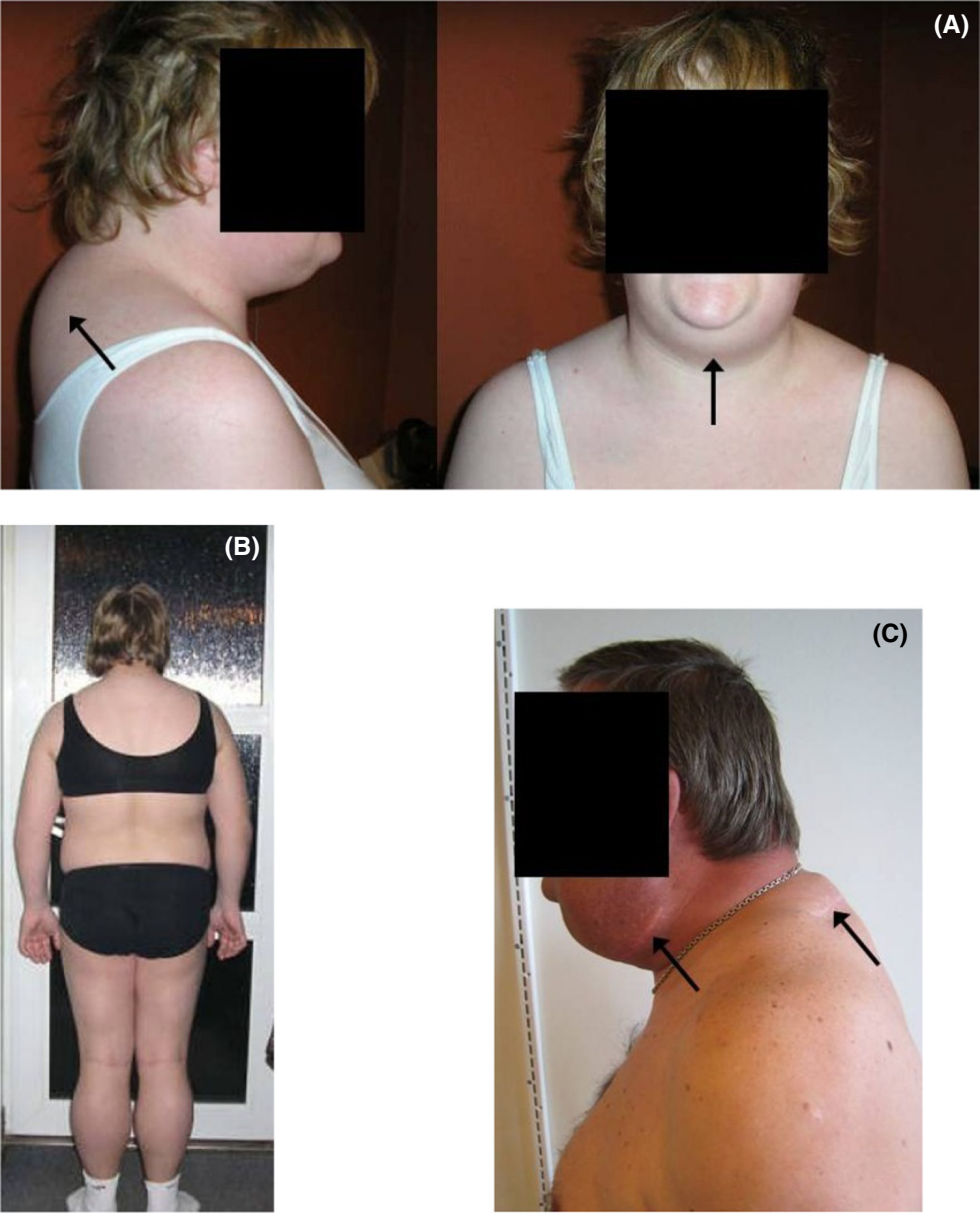
Photographs of the patients with the 8344A>G point mutation of mtDNA. The pictures show the proband aged 27 (A), her younger sister (age 17) (B), and her maternal uncle (age 52) (C). Black arrows indicate the presence of lipomas in A and C, and also the presence of scars in C.

The study included five maternally related family members: Three sisters, their mother, and their uncle (Table 1). The proband (the eldest sister) was originally admitted to our neuromuscular clinic for diagnostic purpose due to symptoms of reduced mental abilities and premature fatigue. Additionally, during the last 2 years prior to admission, she had begun developing lipomas in the upper back and submandibular regions (Fig. 1A). The second oldest sister also suffered from reduced mental abilities and had a masculine appearance (broad shoulders, large hands and feet, and hair growth on her upper lip; see Fig. 1B) indicating an endocrine dysfunction. The youngest sister was asymptomatic, and diagnosis was carried out due to concerns from her and her mother. Their mother was asymptomatic, while their maternal uncle complained of premature fatigue and had numerous, large lipomas in the submandibular and upper back regions. For cosmetic reasons, these lipomas had been partially removed by surgical excision (Fig. 1C) before admitted to our neuromuscular clinic.

**Table 1:** Demographic, morphologic, and biochemical findings in each family member

	Proband	Sister I	Sister II	Mother	Uncle
Age (years)	27	17	14	46	52
Height (cm)	165	171	171	175	173
Weight (kg)	74	100	53	65	86
RRF (%)	10	0	0	<1	0
Cox^−^ fibers (%)	90	0	0	<1	<1
CNF (%)	4	<1	0	0	<1
ICF (%)	Heavy	Minor	None	Minor	Minor
Resting lactate (mmol/L)	7.1* (2.3)	3.4* (2.3)	1.9 (2.3)	3.1* (2.3)	2.1 (2.3)
Peak lactate (mmol/L)	15.5* (11.8)	9.6 (11.8)	5.2 (11.8)	12.8* (11.8)	8.5 (11.8)
P‐CK (mmol/L)	344	123	58	231	5
CS (mU/mg protein)	301	89	119	87	96
CI/CS	0.05†	0.47	0.36	0.23	0.10†
CII/CS	0.36	0.43	0.41	0.31	0.31
CIII/CS	0.29†	1.54	1.82	1.03	0.78
CIV/CS	0.5†	4.5	4.7	2.3	1.3†

Sister I = the second oldest sister, Sister II = the youngest sister, RRF = ragged‐red fibers, CNF = centrally nucleated fibers, ICF = intracellular fat accumulation, P‐CK = plasma creatine kinase, CS = citrate synthase, CI‐CIV = mitochondrial enzyme complexes I‐IV. Activities of the mitochondrial enzyme complexes are corrected for CS activity, and are thus expressed as ratios. Normal ranges for these ratios are expressed as mean ± two standard deviations (2SDs). *Higher than the upper limit of plasma lactate measured in 20 healthy subjects. ^†^Activity lower than 2SDs from the mean. The numbers in parenthesis in row 10 and 11 denotes the upper reference level for resting plasma lactate (row 10) and peak exercise lactate level (row 11).

The patients were seen in our neuromuscular clinic for diagnostic purpose, and for this reason, the study did not need an approval from an ethical committee.

### Investigations

#### Clinical examination

All patients had a neurological examination including muscle strength assessment by Medical Research Council (MRC) scale.

#### Genetic investigations

In all five subjects, the load of the m.8344A>G point mutation was investigated in skeletal muscle, blood, and urine‐ and buccal epithelial cells. In the proband and the uncle, mtDNA mutation load was assessed in apparently unaffected fat tissue and from lipomas. The biopsy, from apparent unaffected fat tissue, was taken from abdominal adipose tissue, and the biopsy of lipoma was taken from the submandibular region/chin. MtDNA mutation load was assessed via methods previously described 16, 17.

#### Biochemical investigations

Plasma creatine kinase (CK) levels and mitochondrial enzyme complex I‐IV and citrate synthase (CS) activities in muscle were measured in all patients according to methods previously described 18.

#### Morphological investigations

Serially cut muscle sections were evaluated for number of internally nucleated fibers, ragged‐red fibers, COX negative fibers, and fibers with fat infiltration with standard staining methods.

#### Functional testing

Maximal oxidative capacity (VO_2max_) and plasma lactate levels at rest and peak exercise were measured in all patients according to methods previously described 19. The findings were compared to that found in 20 healthy sedentary control subjects (10 men and 10 women) presented in Jeppesen et al., 2006 1.

## Results

Clinical examination revealed generalized reduced muscle strength in the proband (4), the second oldest sister (4+) and the uncle (4+).

**Figure 2:**
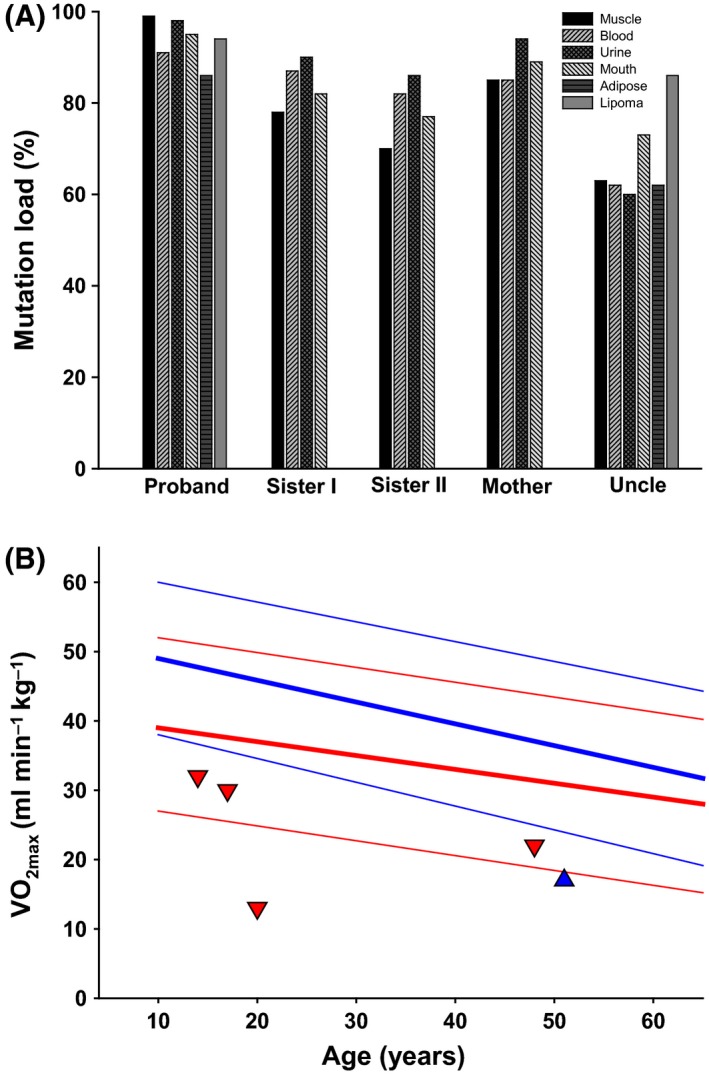
Load of the m.8344A>G mutation among different tissues in each of the five family members (A) and maximal oxidative capacity (VO_2max_) of each family member as determined by cycle ergometry testing (B). (A) Each bar represents a tissue as explained by the legend box. The tissues investigated are the vastus lateralis muscle (muscle), urine epithelial cells (urine), buccal epithelial cells (mouth), leukocytes (blood), clinically unaffected adipose tissue from the abdominal region (adipose), and lipoma tissue from the upper back region (lipoma). Samples from the latter two tissues were only collected from the proband and the uncle as they were the only family members with lipomas. Sister I = the second oldest sister, Sister II = the youngest sister. (B) Corresponding values from healthy, age‐matched, male (in blue) and female (in red) subjects are plotted for comparison. The solid lines indicate the mean value. Broken lines indicate two standard deviations above and below the mean. Sister I = the second oldest sister, Sister II = the youngest sister.

Genetic investigation revealed the m.8344A>G point mutation in all family members. The mtDNA mutation load level was 60–99% in the investigated tissues (Fig. 2A). The intrapersonal levels in mtDNA mutation load were rather uniform in blood, skeletal muscle, urine epithelial cells, and buccal mucosa. In the uncle, there was a large difference between apparent healthy adipose tissue (63%) versus lipoma (87%), while the difference between apparent normal adipose cells and lipomas was less pronounced in the proband (87% vs. 95%).

Activities of mitochondrial enzyme complexes I and IV were reduced in the proband and the uncle, and in addition complex III was impaired in the proband (Table 1).

VO_2max_ was 73% lower in the proband and 54% lower in the uncle compared to age‐ and gender‐matched healthy subjects (Fig. 2B). However, the uncle stopped exercising prematurely due to pain in the right knee. Thus, the VO_2max_ may be “false” low, since the uncle did not reach exercitation. VO_2max_ may therefore be underestimated in the uncle. The VO_2max_ of the remaining family members was low within the normal range (Fig. 2B). Only the proband had elevated plasma CK level, while resting plasma lactate levels were elevated in the proband, the second oldest sister, and the mother. At peak exercise, lactate was elevated in the proband and the mother (Table 1).

Morphological investigations revealed abnormal muscle morphology in the proband while muscle morphology was normal in the rest of the family members (Table 1).

## Conclusions

In this study, we report the pattern of mtDNA mutation load in mitotic‐ and postmitotic tissues in five maternally related family members harboring the m.8344A>G point mutation with presentation of multiple lipomas in two of the family members. We found a uniform distribution of mtDNA mutation load among tissues, except for lipomas in one family member, where mtDNA mutation load was much higher in lipomas cells compared to apparent normal adipose tissue.

Previous case studies that have described patients with lipomas carrying the m.8344A>G point mutation have found that lipomas seem to develop over time 6, 7, 8, 9, 10, 11, 12, 13, 14, 15, and patients have presented with lipomas at middle age like the uncle in this study. In contrast, the proband presented with lipomas in her twenties. We have previously shown, that when tested in the same tissue, there is a close relationship between mtDNA mutation load and tissue phenotype, which indicates that symptoms emerge when mtDNA mutation load reaches a certain level in the tissue 1, 19. At which mtDNA mutation level lipomas develop from adipose cells are unknown, but the mtDNA mutation threshold was apparently exceeded in the adipose tissue situated submandibular in the uncle and the proband. We have no explanation for the differences in mtDNA mutation load in apparent healthy adipose tissue at abdomen versus submandibular lipomas especially in the uncle, where the difference was large. Factors, besides mtDNA mutation load, determining whether lipomas develop or not, could be site‐specific genetic background that has been found in adipose tissue 20. This mechanism is pure speculation and needs to be addressed in future studies. In the proband, the difference between mtDNA mutation load in apparent normal adipose tissue versus lipomas was much smaller than that seen in the uncle, which may relate to a ceiling off effect due to the high mtDNA mutation load levels.

We have previously shown, that the mtDNA mutation level at which oxidative impairment emerge in skeletal muscle tissue is 55–65% in patients with the common m.3243A>G point mutation of mtDNA 1, 19 and 40–50% in patients with large‐scale deletion of mtDNA 19. In this study, the proband had the highest mtDNA mutation load in skeletal muscle compared to the four other family members. In line with the high mtDNA mutation load, the proband was more affected in skeletal muscle (pathologic findings on muscle biopsy, increased CK level, increased plasma lactate, impaired mitochondrial respiratory complex activity, and reduced VO_2max_) versus the rest of the family members. Due to the high level of mtDNA mutation load in all tissues investigated (>60%) and due to the low number of patients (*n* = 5), it is not possible to establish a mtDNA threshold, at which patients with the m.8344A>G mutation develop symptoms of skeletal muscle tissue. The second eldest sister and the mother had normal VO_2max_, CK, muscle morphology, and mitochondrial respiratory complex activity despite high mtDNA mutation load in skeletal muscle (80% and 85%). These data indicate that the threshold level for mtDNA mutation load, at which oxidative capacity becomes impaired, is higher in patients with the m.8344A>G mutation than in patients with the m.3243A>G mutation.

Studies have showed that mtDNA mutation load level in mitotic tissue decrease over time in patients with the m.3243A>G point mutation 2 and single large‐scale deletion of mtDNA 21 and accumulate over time in postmitotic tissue in patients with single large‐scale deletions 22. These findings have prompted the idea, that there is a postnatal modification of mutation load in patients with mtDNA mutations, which is determined by the mitotic dynamics of the tissue. In this study, the intrapersonal mtDNA mutation load was quite similar among the tested tissues, which indicates that the pattern of segregation is different in the m.8344A>G mutation.

In conclusion, this study demonstrates a highly variable intrafamilial phenotype in persons carrying the m.8344A>G mutation, despite an overall uniform intrapersonal distribution of the mutation load in various tissues, except in adipose tissue in one patient. The difference between mtDNA mutation load between abdominal and submandibular adipose cells may relate to site‐specific differences in genetic background and mtDNA mutation load.

## Consent

The patients presented in this study have given their written informed consent for publication of this Full Case Report and accompanying images.

## Conflicts of Interest

None of the authors had any competing interests in the study.

## Authorship

TDJ: performed clinical examination and exercise test of the patients, carried out muscle biopsy and collected tissue samples for genetic and biochemical testing, performed data/statistical analysis, and contributed to the writing process. NAH: performed exercise test of the patients, collected tissue samples for genetic testing, performed data/statistical analysis, and contributed to the writing process. MD: performed genetic analysis and statistical analysis of the data and made critical revision of the text and figures. FW: performed biochemical analysis of mitochondrial respiratory complex activities, performed statistical analysis of the data, and made critical revisions of the text and figures. GA: performed exercise test of the patients, collected tissue samples for genetic testing, and made critical revisions of the text and figures. JV: performed critical revision of data, figures, table, and text and coordinated and supervised the writing process.
